# Online psychological therapy for kids during social distancing: A study case in a brazilian clinical setting

**DOI:** 10.1192/j.eurpsy.2021.800

**Published:** 2021-08-13

**Authors:** M. Campos, C. Varanda

**Affiliations:** 1 Institute Of Human Sciences - Faculty Of Psychology, Universidade Paulista, SANTOS, Brazil; 2 Human Sciences, Universidade Paulista, Santos, Brazil

**Keywords:** social distancing, online therapy, children’s psychological therapy

## Abstract

**Introduction:**

In response to the spread of COVID-19, many Brazilian therapists faced the challenge of taking their practices online considering legal and ethical issues, besides learning to handle new technologies in a way the therapeutic setting was maintained. The cooperation of the family is fundamental for the creation and maintenance of an adequate therapeutic setting. Children are not sufficiently mature to speak clearly about what bothers them or to talk about how they feel and why, so, drawing, pretend playing, story telling, playing games are the common tools for children’s communication during therapy.

**Objectives:**

Evaluating if online therapy for children can support therapeutic play tools and be effective in a virtual environment preserving the therapeutic setting.

**Methods:**

Two children aged 6 to 11 attended the psychological sessions that were conducted through video calls.The family should provide a silent and private room for those sessions. The children were free to choose the toy they would like to play with and that was available at home such as board games, comic and story books. Mimicry, drawing, an adaptation of the Winnicott Squiggle Game were used, as well as electronic games through screen sharing.

**Results:**

The emotional conflicts were expressed either through conventional games and play or electronic games. Playing with children online was possible as well as maintaining the therapeutic alliance in order to carry on with the treatment in a proper therapeutic setting.

**Conclusions:**

Online therapy for kids showed to be an effective form of service delivery, under strict measures of social distancing in Brazil.

